# Some Personal Reflections

**Published:** 1883-01

**Authors:** 


### SOME PERSONAL REFLECTIONS.

The present issue closes the third year of the publication of the ANNALS OF ANATOMY AND SURGERY, as a monthly periodical, and it seems to be a favorable time for indulging some reflections as to the reason of its being, and the aims of its future. It still remains, as when instituted, the only journal devoted to Surgery, and to Anatomy as related to Surgery, published in the English language. The first number of the *Revue de Chirurgie*, which appeared in France nearly at the same time, added a similar journal for the use of those familiar with the French language—"Langenbeck's *Archives*," "Volkman's *Series of Clinical Lectures*," the *Centralblatt*, and the *Zeitschrift für Chirurgie*, have continued to present to readers of German the surgical work of that nation. That in America also should be maintained a special journal of Surgery, there is found ample reason in the number and quality of the special workers in this field, in the character of the problems which engross its students, and in the rapid strides towards scientific precision, which surgical practice has witnessed within the past two decades. It is true that much of a surgical character appears in the general medical journals of this country, and especially in the great weeklies. These afford a specially favorble field for the first announcement of any step or fact, that its wide diffusion may be early secured, but in the crowding of the weeks upon each other, and in the variety of the interests which the columns of the weeklies must reflect, they cannot be so well adapted for elaborate articles, nor for presenting matter in the way best adapted for preservation and reference. The general weekly journal, moreover, can give to new publications nothing more than cursory notices that are of no value, either in aiding a reader to form an opinion as to the character of the work noticed, or to keep himself informed of the advances in knowledge that it may evince. The long intervals at which a quarterly appears, are too great for the interest in it to be sustained from one number to another.

However, it may once have been, a monthly or a quarterly which now attempts to include in its compass the whole of medicine, is at once crippled by the size of the field, but if it restricts itself to a special department, it presents the happiest union of the elements needed for the best presentation of whatever may be of permanent value in that department. The specialization of journals is thus, but the natural result of that specialization of study and practice which is the inevitable result of the growth of knowledge. The power of this tendency to specialization has shown itself in the gradual development of this journal from being the simple record of a special local society, to a point where it may presume to claim consideration as an exponent of American Surgery in general. In order to favor the future development of the journal as a general exponent of Anatomy and Surgery, it has been thought best that it should not be, even apparently, the organ of any one society, and accordingly the Anatomical and Surgical Society of Brooklyn, by whose direction and support it was inaugurated, have relinquished their special interest in it, and henceforth its editors will be solely responsible for its contents and character.

On the eve of the beginning of a new year, the editors take the opportunity of announcing their aim to continue the practical and high character of the contents of the journal, and to make its tone progressive and liberal. They will have the assistance of the same corps of associate editors as in the past, with the addition of Dr. Roswell Park, of Chicago, Lecturer on Surgery in Rush Medical College, and surgeon to the Michael Reese Hospital. The *Annals of Anatomy and Surgery* will therefore continne to be issued monthly, and will present, in each number, detailed studies and elaborate articles from special contributors, and careful editorial reviews, digests and discussions of such topics and books as may seem to be of special importance, from time to time. Articles for the journal are now in course of preparation by Prof. W. T. Briggs, of Nashville, Tenn.; C. B. Keetley and R. J. Godlee, Esqs., of London, Eng.; Dr. H. D. Schmidt, of New Orleans, La.; Profs. Moses Gunn and Edmund Andrews, of Chicago, Ill.; Prof, W. W. Keen, of Philadelphia; Prof. Hunter McGuire, of Richmond, Va.; Dr. H. A. Martin, of Boston, Mass.; Prof. F. H. Gerrish, of Portland, Me.; Dr. F. W. Rockwell, of Brooklyn, N. Y.; Profs. T. F. Prewitt and

L. Bauer, of St. Louis, Mo.; Prof. P. S. Conner, of Cincinnati, O.; Prof. Randolph Winslow, of Baltimore, Md.; Drs. H. Banga and W. T. Belfield, of Chicago, Ill.; Prof. A. L. Ranney, of New York; and Prof. Wm. Osler, of Montreal, Canada.

The scope of the journal will include also, as indicated by its title, particularly those lines of anatomical research which have a bearing upon practical surgery.

The scholarly sketches of the old masters of Anatomy and Surgery, which for three years have regularly appeared in the journal, from the pen of Dr. George Jackson Fisher, of Sing Sing, N. Y., will be continued during the year to come. Beginning with Avicenna in the January number, Hally Abbas, Albucasis, and the school of Salernum will successively form the subjects of sketches.

During 1883, in addition to the features which have heretofore characterized the journal, it will publish the proceedings of the New York Surgical Society, which will include many elaborate papers and discussions of the greatest value.

Finally, whatever shall promise to make the journal more completely representative of American Surgery, or more valuable to American surgeons, it will at all times be the effort of its editors to adopt.—*Annals of Anatomy and Surgery.*

